# The Predominance of the Clinician

**Published:** 1908-04-25

**Authors:** 


### The Predominance of the Clinician.

That lively depicter of the contemporary social scene, the Hon. G. W. E. Russell, lately drew a silhouette of the young London consultant, contrasting him favourably with the Sawyers, Allens, and Huxters of Dickens's and Thackeray's day. Well-bred instead of a grosser sort of Bohemian; fond of his art, and yet, as a good cricketer or golfer, mindful of the " gymnastic " as well as of the " music "; given to reading, if not the classics of literature and science, at any rate present-day authors whose appeal is to intelligent people, he marries, as such a model 'prentice should, the daughter of one of his seniors, and the last impression given is of his introduction into the good graces of the most influential of his father-in-law's patients.

Now although the observation is welcome that medical progress and general intellectual expansion have left their mark on the *personnel* of our profession, one may be excused for seeing in the tone of the above appreciation an indication that something still remains to be achieved. There is quite a suggestion that the highest state of the medical man is to sign bulletins and to keep himself before the eye of the public as a fashionable physician. Perhaps there are reasons why a layman should think so. For many the greatest triumph of medical science is that impressive response to individual feelings of consternation or compassion which a first-class clinician's skill often enables him to make. If this be analysed, however, there is surprisingly little in it; if (unkindly) compared with the faculty of a Harvey or a Hunter, nothing at all. An accomplished clinician possesses keen and refined senses, strong judgment, tenacity, and that " clinical instinct " which is probably the power of original observation in a much attenuated form. But although one certainly must not say of his work, as Dr. Johnson did of a much-discussed expedition, that there is " very little of intellectual about that course "; still no one will be likely to maintain that a first-rate mind can find its reasoning powers fully exercised by diagnosis and treatment, however advanced the methods used. The deep satisfaction in clinching various doubtful matters with one rapid, but thorough and certain, physical examination, gratifying though it is, is yet no purely intellectual pleasure. John Hunter, indeed, to whom reference has been made, plied his clinical work mainly as a means of getting funds for research, achieving in those early days a double success which it would take an even greater mental giant to equal in this more sophisticated twentieth century. As to the clinician's immediate usefulness there can be no question; on the other hand, the classic triumphs of preventive medicine have to be dinned into the public ear continually in order that a little money may be got for experiments which are urgently required, but which may, through nobody's fault, turn out quite nugatory. All this is a very large and complex matter, and one not to be lightly approached, since much may be said for the existing order of things. There can be no harm, however, in noting some of the anomalies involved, removal of which would probably do more than anything else to raise the doctor's status. At present the laity show too much disposition to regard him merely as the chief of a sick person's train of attendants. The title of certain German congresses announces them as for " Naturforscher und Aertzte ": the profession will gain largely in importance when, owing to current tendencies shown in the gradual supplanting of humanism by science and in certain economic movements, this distinction becomes no longer possible.

